# Painful Terminal Neuroma Prevention by Capping PRGD/PDLLA Conduit in Rat Sciatic Nerves

**DOI:** 10.1002/advs.201700876

**Published:** 2018-03-27

**Authors:** Jiling Yi, Nan Jiang, Binbin Li, Qiongjiao Yan, Tong Qiu, Killugudi Swaminatha Iyer, Yixia Yin, Honglian Dai, Ali K. Yetisen, Shipu Li

**Affiliations:** ^1^ State Key Laboratory of Advanced Technology for Materials Synthesis and Processing Wuhan University of Technology Wuhan 430070 China; ^2^ School of Molecular Sciences University of Western Australia 35 Stirling Hwy Crawley WA 6009 Australia; ^3^ School of Engineering and Applied Sciences Harvard University Cambridge MA 02138 USA; ^4^ Brigham and Women's Hospital Harvard Medical School Cambridge MA 02115 USA; ^5^ School of Chemical Engineering University of Birmingham Edgbaston Birmingham B15 2TT UK

**Keywords:** inflammation, nerve conduits, neuroma prevention, painful scar neuropathy, scar deposition

## Abstract

Neuroma formation after amputation as a long‐term deficiency leads to spontaneous neuropathic pain that reduces quality of life of patients. To prevent neuroma formation, capping techniques are implemented as effective treatments. However, an ideal, biocompatible material covering the nerves is an unmet clinical need. In this study, biocompatible characteristics presented by the poly(D,L‐lactic acid)/arginylglycylaspartic acid (RGD peptide) modification of poly{(lactic acid)‐*co*‐ [(glycolic acid)‐alt‐(L‐lysine)]} (PRGD/PDLLA) are evaluated as a nerve conduit. After being capped on the rat sciatic nerve stump in vivo, rodent behaviors and tissue structures are compared via autotomy scoring and histological analyses. The PRGD/PDLLA capped group gains lower autotomy score and improves the recovery, where inflammatory infiltrations and excessive collagen deposition are defeated. Transmission electron microscopy images of the regeneration of myelin sheath in both groups show that abnormal myelination is only present in the uncapped rats. Changes in related genes (MPZ, MBP, MAG, and Krox20) are monitored quantitative real‐time polymerase chain reaction (qRT‐PCR) for mechanism investigation. The PRGD/PDLLA capping conduits not only act as physical barriers to inhibit the invasion of inflammatory infiltration in the scar tissue but also provide a suitable microenvironment for promoting nerve repairing and avoiding neuroma formation during nerve recovery.

## 1 Introduction

More than 185 000 limbs are amputated in the United States annually.^1^ In 2005, the prevalence of limb loss was 1.6 million, which is projected to double by 2050.^2^ Besides the loss of a body part and its motor function, the majority of patients experience certain level of painful sensation after wound healing. These shock, burning, or electrical‐like pains can persist from months to years and impair patients' life quality.^3^ This unsatisfied feeling is aroused by the occurrences of neuromas distributed at the stump of injured nerve.^4^ For the initial damage in peripheral nerve, residual axons undergo Wallerian degeneration, apoptosis, axons extension, and remyelination to achieve recovery.^5^ However, this limits regenerative ability that is not sufficient to overcome the long‐distance gap (>50 mm) or fail without the guidance of distal stump, which are typical neuropathic symptoms in serve trauma and amputation.^6^ In this case, a sprout can be observed at the proximal stump and axon extends spontaneously,^7^ but they are randomly oriented and chaotic milieu triggers a subsequent disorganization of remyelinations. Finally, a bulbous tissue (neuroma) is formed.^8^ Within the neuroma, many regenerated axons are surrounded by abnormal myelin sheath, exhibiting variable degrees of thickening, which alters the electrophysiological properties of axons and renders them hypersensitive to mechanical, chemical, and physical stimuli.^9^ Even worse, this hyperpathia can intensify by the factors that release at the stage of immune reaction, such as monocyte chemoattractant protein‐1, interleukin‐1 (IL‐1), and tumor necrosis factor‐alpha (TNF‐α).^10^ In prolonged inflammation, collagen and mature myofibroblasts may infiltrate into the neuroma, which acts as the main source of mechanical irritation on regenerating nerves, subsequently generating a persistent painfulness.^11^

Surgical excision is performed as an acceptable treatment of neuroma at early stage. The terminal nerve stump is shortened to a non‐neuroma site, but a new neuroma is prone to relapse at the new lesion site.^12^ A common practice is that the axons are buried into adjacent tissue, such as bone, muscle, or vein.^13^ However, this surgery is not suitable for digital nerve, because muscles in hand are small and display respectably excursions during body movement, which lead to a direct mechanical contraction or traction on nerve.^14^ Therefore, utilizing nerve conduit to bridge or cover injured nerve has been introduced as a promising approach. Since then, the nerve graft has become the gold standard for nerve reconstruction and neuroma prevention, while the donor site morbidity and immunogenic host response remained a challenge.^15^ Galeano et al. have first utilized the host's femoral vein and this procedure fundamentally avoided the immunogenicity during protecting the lesion site against external physical stimulation and inflammation and neurotrophins overstimulating the nerve fiber regeneration.^16^ The elasticity of femoral vein allowed capping nerve fibers by microsurgery suturing. Although the host's femoral vein was biocompatible and it mechanically isolated the transected nerve stump from the environment that exerted a preventive effect on neuroma formation, this approach was limited since grafting femoral vein added a layer of surgical complexity, which was not practical for clinical applications.^17^ Therefore, “ready‐to‐use” artificial capping conduits were gradually developed. For instance, Bertleff et al. have developed a resorbable poly(D,L‐lactide‐caprolactone) nerve capping conduit for the treatment of neuromas.^18^ The developed conduit decreased axonal sprouting and lowered the risk of adhesion between the scar tissue and sensory reinnervation of overlying skin. No adverse effect of capping conduit graft was observed in four patients enrolled in this study.^18^ However, biocompatibility, swelling, degradation rate, and automutilation issues were recorded in recent animal and clinical studies.^19^ Hence, the performance of artificial capping conduits should be improved to match the effectiveness of autologous nerve graft.

Recently, a biocompatible RGD‐functionalized artificial conduit was synthesized for nerve regeneration, in which no neuroma was formed after a 5 week implantation. Additionally, the functionalized RGD peptide showed the ability to attenuate inflammation reaction, which was crucial for painful neuroma development.^20^ Here, an RGD functionalized PRGD/PDLLA capping conduit was developed to inhibit neuroma formation. To assess the feasibility of this approach, nerve terminal resection models were established by removing rat distal ends of sciatic nerve and the proximal stumps were capped by PRGD/PDLLA conduits. Changes in behavior, histological results, and the potential molecular mechanism were analyzed to evaluate the therapeutic efficacy of PRGD/PDLLA capping conduit in reducing traumatic neuroma formation.

## 2 Results

### 2.1 Autotomy and Pain‐Related Markers Assessment

**Figure 1:**
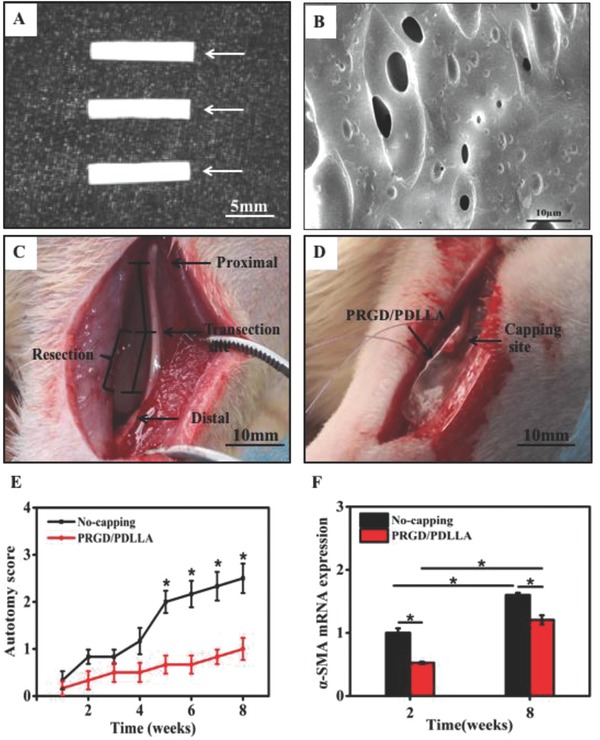
Surgical procedures of the transected rat model and autotomy assessment. A) PRGD/PDLLA conduit and B) surface morphology observation using scanning electron microscopy. C,D) Transected rat model, the right sciatic nerve was transected into proximal and distal segments at the center of the right thigh, the proximal stump was capped by a PRGD/PDLLA capping conduit and the distal stump was removed for 10 mm to avoid spontaneous nerve regeneration. E) The assessment of weekly average autotomy score from 1 to 8 weeks postoperatively (*n* = 6, *P < 0.01, compared to a noncapped group). F) The expression level of α‐SMA mRNA in different groups at 2 and 8 weeks postoperatively, noncapped group as the control (*n* = 3, *p < 0.01).

The morphology of PRGD/PDLLA capping conduit and surgical procedure in the rat model are illustrated in **Figure** **1**A–D. The autotomy behaviors were observed on the operated areas in all animals and the average autotomy scores were recorded once a week between the PRGD/PDLLA capped and noncapped groups (Figure 1E). The occurrence of autotomy in PRGD/PDLLA capped group was lower than that in noncapped group and exhibited significantly statistical differences (*P* < 0.01), especially from the 5th to the 8th week. The average autotomy score in PRGD/PDLLA capped conduit group was 1.0 ± 0.2, while the average score of noncapped group (2.5 ± 0.3) was a nearly two times higher value. The trend of α‐SMA expression was mainly consistent with the autotomy score assessment. As a neuropathic pain related marker, quantitative real‐time polymerase chain reaction (qRT‐PCR) results indicated that its transcriptional level was distinctly lower in PRGD/PDLLA capped group (respectively 0.53 × and 1.21 ×, *P* < 0.01) as compared to noncapped group (respectively 1.0 × and 1.6 ×, *P* < 0.01) at both intervals (Figure 1F).

### 2.2 Inflammation Reaction

**Figure 2:**
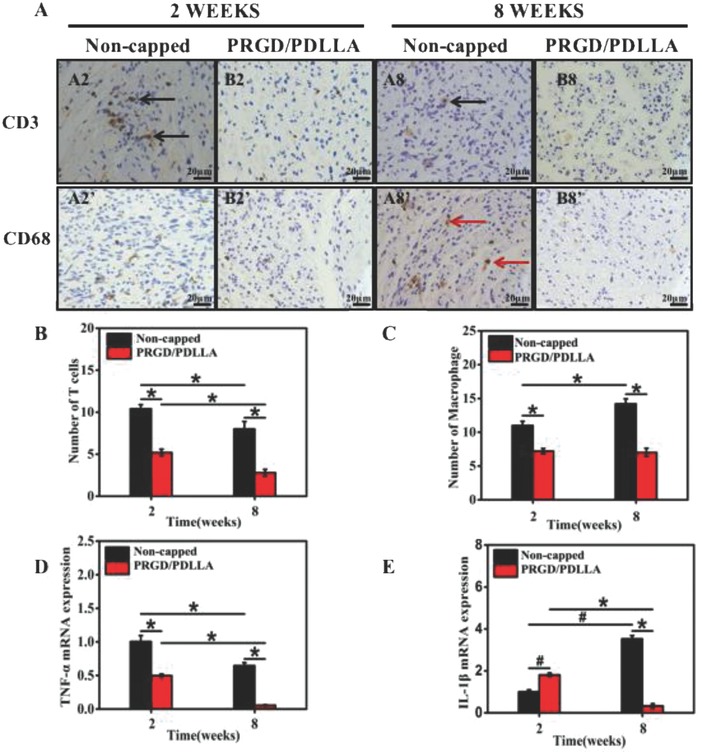
Cross‐sections of the proximal nerve stumps with immunohistochemical staining at both 2 and 8 weeks postoperatively. A2–B8) Labeled with immunohistochemical antibodies of CD3 (black arrow). A2'–B8') Labeled with immunohistochemical antibodies of CD68 (red arrow). B,C) The statistics of T cells (CD3) and macrophages (CD68) in different groups. D,E) The mRNA expression of TNF‐α and IL‐β in different groups (*n* = 6, *P < 0.01, # *p* < 0.05, compared to noncapped group).

Immunohistochemistry was employed to determine the degree of inflammation infiltration at the lesion site, in which T cells and macrophages were analyzed (**Figure** **2**A). The number of T cells in both groups was decreased at the 8th week. However, the number of T cells in noncapped group (control group) (10.4 ± 0.5 and 8.0 ± 0.5, *P* < 0.01) was significantly larger than that of PRGD/PDLLA capped group (5.2 ± 0.4 and 2.8 ± 0.4, *P* < 0.01; Figure 2B) at both intervals. No obvious change could be observed in the macrophages in PRGD/PDLLA capped group from the 2nd to the 8th week (7.2 + 0.4 and 7.0 ± 0.6, *P* > 0.05). On the contrary, the number of macrophages in noncapped group was increased from the 2nd to 8th weeks (11.0 ± 0.6 and 14.0 ± 0.8, *P* < 0.01) and was higher than PRGD/PDLLA capped group (Figure 2C). Inflammatory cytokines of TNF‐α and IL‐1β were detected by qRT‐PCR analysis (Figure 2D,E). The expression of TNF‐α in both groups reduced at 8 weeks, but TNF‐α expressed in noncapped group (1 × and 0.6 ×, *P* < 0.01) was distinctly higher than PRGD/PDLLA capped group (0.5 × and 0.06 ×, *P* < 0.01), respectively, at both time intervals. The expression of IL‐1β demonstrated a different pattern. A higher level of IL‐1β was detected in PRGD/PDLLA conduit group at the 2nd week and subsequently downregulated (1.8 × to 0.3 ×, *P* < 0.01). In noncapped group, IL‐1β was upregulated (1 × to 3.5 ×, *P* < 0.05).

### 2.3 Collagen Deposition and Scar Formation

**Figure 3:**
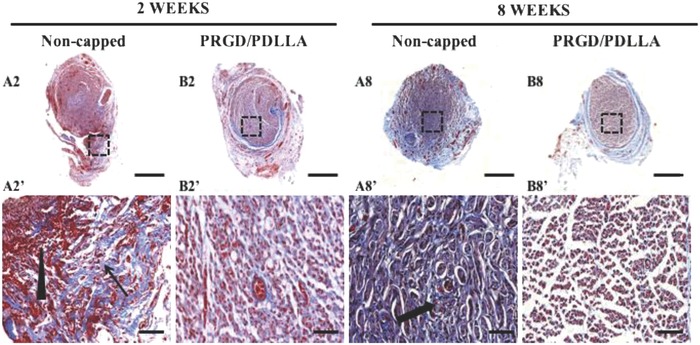
Cross‐ sections of the proximal nerve stump with Mason's trichrome masons staining at 2 and 8 weeks postoperatively. The collagen deposition, clotted blood, and disorganized nerve fibers are indicated by the thin arrow, triangle, and thick arrow, respectively. (A2–B8, scale bar: 500 µm; A2'–B8' is the partial magnified view of A2–A8, scale bar: 50 µm.)

Masson's trichrome staining showed that the nerve fibers in noncapped group were arranged irregularly and haphazardly, appearing with an expansion of collagen deposition (blue) and clotted blood (red) at 2 weeks (**Figure** **3**A2,A2'). After 8 weeks, a large amount of small and haphazardly arranged nerve fascicles was mingled with a dense collagen tissue (Figure 3A8,A8'). In the PRGD/PDLLA capped group, nerve fibers maintained a clear and regular morphology. Most fibers had normal size and were arranged as insulated pattern, although small swells were also observed at the 2nd week. Average diameters of nerve cross section shrunk from 2 to 8 weeks.

**Figure 4:**
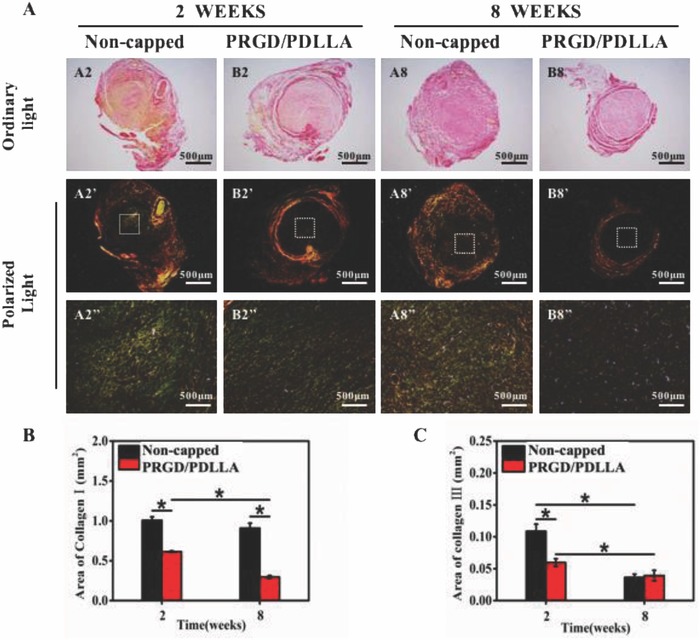
Sirius red staining and analysis of proximal nerve stumps at 2 and 8 weeks postoperatively. A) Images of sirius red staining, collagen I (thick fibers) presents yellow orange; collagen III (thin fibers) presents green. (A2''–B8'' is the partial magnified view of A2'–A8'.) B) The average area of collagen I in different groups. C) The average area of collagen III in different groups (*n* = 5, *P < 0.01, compared to noncapped group).

Sirius red staining was utilized to evaluate collagen formation and distribution by polarized light microscopy (**Figure** **4**A). Results showed that two types of collagen coexisted at nerve stump (collagen type I: thick, yellow or red fibers; collagen type III: thin, greenish fibers). The content of collagen I in noncapped group (1.0 ± 0.04 mm^2^ and 0.91 ± 0.06 mm^2^) was significantly higher than that of PRGD/PDLLA capped group (0.61 ± 0.01 mm^2^ and 0.30 ± 0.02 mm^2^, *p < 0.01) in both intervals (Figure 4B). For collagen III (Figure 4C), its content was lower, and the region of collagen III in PRGD/PDLLA capped group (0.06 ± 0.006 mm^2^, *p < 0.01) was still smaller than that of noncapped group (0.11 ± 0.01 mm^2^,* *p* < 0.01) while it was downregulated and similar values were achieved in both groups at the 8th week.

### 2.4 Demyelination

**Figure 5:**
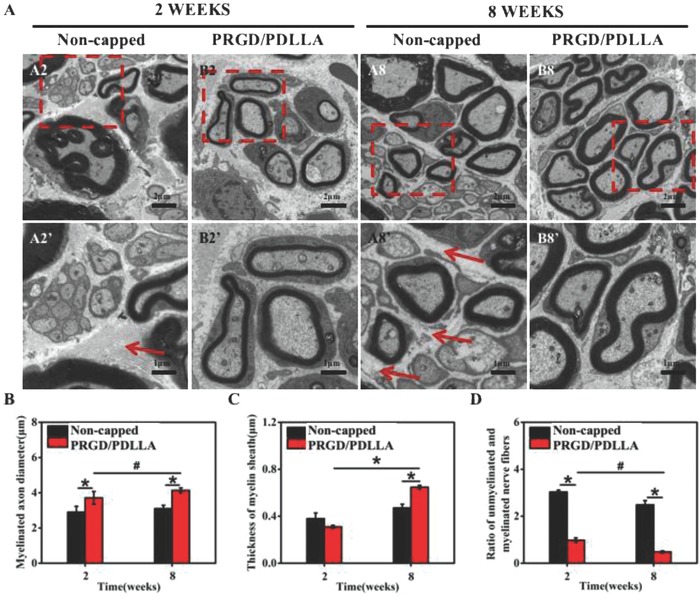
Observation and assay of cross‐sections of the proximal nerve stump at both 2nd and 8th weeks. A) Morphology of myelin sheath in different groups (A2'–B8' is the partial magnified view of A2–A8) observed by TEM. B–D) Myelinated nerve fiber diameter, myelin sheath thickness, and the ratio of unmyelinated and myelinated nerve fiber were quantitatively evaluated and statistically analyzed. The red arrows show the collagenous fibers with larger particles (*P < 0.01, ^#^ *P* < 0.05, compared to noncapped group).

Structural differences of nerve fibers in each group were compared using transmission electron microscope (TEM) imaging to evaluate effects of PRGD/PDLLA capping conduits on remyelination (**Figure** **5**A). Micrographs within an area of 10 µm^2^ were randomly selected for analysis. The ratio of unmyelinated and myelinated fiber, diameter of myelinated fiber, and thickness of myelin sheath were measured from the selected micrographs and statistically analyzed. The diameters of axons in PRGD/PDLLA capped group (3.71 ± 0.36 µm to 4.10 ± 0.14 µm) were longer than that of noncapped group (2.90 ± 0.33 µm to 3.10 ± 0.19 µm) at both intervals (Figure 5B). Compared with noncapped group (0.47 ± 0.03 µm), a thicker sheath could be detected in PRGD/PDLLA capped group (0.65 ± 0.02 µm) at 8th weeks (Figure 5C). The ratio of unmyelinated and myelinated fibers in the noncapped group (3.03 ± 0.08 µm) was nearly threefold higher than that of PRGD/PDLLA capped group (0.98 ± 0.10 µm) at the 2nd week. This ratio only decreased in PRGD/PDLLA capped group (0.48 ± 0.04 µm) at the 8th week (Figure 5D). Additionally, numerous collagenous fibers with larger particles (red arrow) were present in the noncapped group.

**Figure 6:**
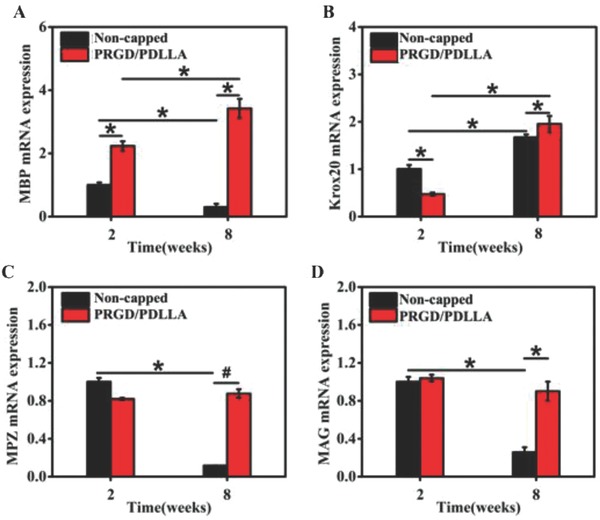
Quantification analyses of the myelin maturation relative genes in different groups. A–D) MBP, Krox20, MPZ, and MAG, respectively (*P < 0.01, ^#^ *P* < 0.05, compared to noncapped group).

To investigate whether the expressions of myelin maturation related genes were changed after capping with PRGD/PDLLA conduit, qRT‐PCR analyses were carried out and genes MBP, Krox20, MPZ, and MAG were tested. **Figure** **6**A shows that MBP was continuously expressed in PRGD/PDLLA capped group in both intervals (2.23 × and 3.42 ×, *P* < 0.01), while the expression of MBP in noncapped group notably decreased at the 8th week (1.00 × and 0.52 ×, *P* < 0.01). For gene Krox20, expression in capped group was slightly lower than control at the 2nd week (respectively 0.47 × and 1.0 ×, *P* < 0.01), while it became higher than noncapped group (respectively 1.95 × and 1.67 ×, *P* < 0.01) at the 8th week (Figure 6B). Similar changing trends were shared by MPZ and MAG (Figure 6C,D), both of these two genes were downregulated in noncapped group (respectively 0.12 × and 0.26 ×) at the 8th week. No obvious regulation was detected in PRGD/PDLLA capped group at both intervals (respectively 1.04 × and 0.9 × for MAG, 0.82 × and 0.86 × for MPZ, *P* > 0.05).

## 3 Discussion

Since the first successful report of peripheral nerve repair in 1836, it has been recognized that not all the nerve injures were amenable to direct end‐to‐end repair as the elasticity was limited.^21^ According to Seddon classifications for nerve injury, the two worst forms of injury were axonotmesis and neurotmesis, which referred to the presence of a gap in the axon or complete severance of nerve trunk.^22^ In these situations, surgical repair was often postponed because of the potential suture line dehiscence caused by delayed necrosis.^17^ Additionally, neuroma might be formed during this interval, which was often accompanied by hyperalgesia, allodynia, and cold intolerance.^23^ Sometimes extreme pain will cause the function loss of remaining part of body and productivity, resulting in high unemployment as well as high personal and public expenditures.^24^ For this painful sequel, a series of treatment principles has been developed.^14^ After the nerve injury, the path‐physiological response of transected nerve such as inflammation factors, growth factors, and scar tissue stimulation leads to neuroma formation, especially the painful neuroma formation at the nerve transection site. A commonly used neuroma prevention approach is shortening; however, its positive effects are very often transitory because of neuroma's natural tendency to reoccur at the new nerve transection site. Covering the stump, which can be achieved by burying the nerve stump into a nearby anatomic structure or by capping it with different materials, isolates the nerve stump from the environment to obtain a preventive effect on neuroma formation.^16^, ^24^ However, high risk of neuroma was formed in the resection.^25^ To address these issues, nerve guidance capping conduits have been developed for the realignment of two stumps of damaged nerve.^26^ Some of these nerve capping conduits have the ability of providing a suitable microenvironment for promoting the neural regeneration.^17^ While different characteristics of the capping materials are needed in clinical applications for different kinds of damaged nerves, especially in amputation, the nerve injury is permanent. Full restoration of function cannot be achieved, due to the absence of whole distal stump.

In the present work, a rat nerve terminal resection model was established and autotomy scoring was employed to evaluate the effects of PRGD/PDLLA capping conduits on pain suffering. As a response to denervation in the sciatic nerve, rats lick, scratch, and self‐mutilate their limb, which could be quantified via a modified Wall scale.^27^ Over 8 weeks of behavioral studies, significant differences in the autotomy were noted between the PRGD/PDLLA capped group and noncapped group. The average score of noncapped group was nearly two times higher than that of PRGD/PDLLA capped group, which implied an alleviation of painfulness in PRGD/PDLLA capped group. However, some studies reported that autotomy score as a pain index was controversial; the positive correlation between them could be interfered by another experimental variable. Animals alone could experience more stress than those housed with cagemates, which would directly increase autotomy.^28^ This maybe also as a result of excessive glooming in the absence of sensory feedback.^29^ These potential factors require further investigation in animal models to evaluate the efficacy of PRGD/PDLLA capping conduit.

The analysis of inflammation reactions in the two groups showed that the role of inflammation for peripheral nerve injury was binary that moderate inflammation was essential during the process of regeneration; however, it was also the main source of pain. At the early stage of inflammation, the release of chemokines and increase in the permeability of blood–nerve barrier were accomplished simultaneously.^30^ Blood‐borne macrophages attracted from surrounding circumstance would traverse these vessels along the gradient and the majority of active macrophages was hematogenous. TNF‐α and IL‐1β were also secreted for nociceptor sensitization. Moreover, the distribution of inflammatory cells and the expressions of cytokines in the noncapped group were different from those in the PRGD/PDLLA capped group. There were more macrophages and T cells invading the lesion site in the rats without treatment, as well as a persisting high level of TNF‐α and IL‐1β. These results were in accordance with the hallmarks of chronic inflammation, in which sustained macrophage influx hindered the resolution of inflammation and maintained the expression of genes of TNF‐α and IL‐1β.^31^ These cytokines in turn increased the blood–nerve barrier permeability and more inflammatory cells would accumulate at the lesion site to exacerbate or perpetuate the inflammatory process.^32^ In the rats with capping conduits, milder infiltration together with downregulation of related genes indicated that the inflammation was suppressed, which was mainly attributed to the presence of PRGD/PDLLA capping conduits. It could directly insulate the overabundant inflammatory cells and RGD peptides incorporation could enable it to inhibit aberrant inflammatory activities. Abundant RGD‐motifs within extracellular matrix was shown to abolish the release of cytokines with the interactions of integrin.^33^ The anti‐inflammation property of PRGD/PDLLA capping conduits was also evidenced by collagen deposition.

Due to the close connection between neuroma formation and fibrosis, histological samples in the two groups (PRGD/PDLLA capped group and noncapped group) were investigated. Without the protection of PRGD/PDLLA conduit, typical characteristics of neuroma could be demonstrated in the control rats: haphazard patterns of regenerated axons and high degree of collagen deposition, which were the sequel of extraordinary fibrosis.[[qv: 33b]] As another essential event for wound healing, the development of fibrosis was supposed to maintain the origin architecture of tissue and provide mechanism support for axons sprouting.^34^ However, the prolonged inflammation would constantly trigger the activation of fibroblast via TGF‐β1, and plenty of collagens was generated during the differentiation of fibroblasts into myofibroblasts.^35^ The histological staining revealed that the collagen I, a major constituent of the fibrous connective tissue, was reduced at 8 weeks, although the expression of collagen III in both groups has not significantly changed at the end of 8 weeks. Since TGF‐β1‐mediated network was an RGD peptide‐integrin‐dependent signal pathway, the subtle collagen deposition was another evidence for the anti‐inflammation property of PRGD/PDLLA capping conduits. Additionally, excessive fibrosis was a second irritation factor of neuroma‐associated pain. During the development of fibrosis, anagenetic axons might be entrapped by myofibroblasts, leading to a long‐lasting mechanical stimulation.^36^ Hence, qRT‐PCR assay was carried out to access the activities of myofibroblasts via α‐SMA. Recently, this indicator was shown to be an indirect biomarker of autotomy behavior.[[qv: 11b]] Similar conclusions could be derived from the present work, changes in its expression and autotomy scoring exhibited the same trend. Although upregulation was verified in both groups, a lower value was measured in the capped group.

Effects of PRGD/PDLLA conduit on remyelination were also evaluated. Conduits were able to defend the nerve fiber, and the axons were protected by myelin within the complicated cable. Myelin sheath wrapped axons in a spiral fashion and acted as an insulator.^37^ Any potential deficiency in myelin would risk axon damage by mechanical pressure, and a cross talk between adjacent nerves might be developed due to the lack of insulation. Although the ectopic impulse generated from dorsal root ganglion was impossible in this rodent sciatic nerve resection model, it is common in the amputation neuroma and allodynia would be arisen by the multiplication of normal nerve impulses triggered by gentle stimulation.[[qv: 4b]] Whenever peripheral nerve injury occurred, the myelin phagocytosis would be accomplished until the last phase of inflammation response. Along with the remyelination progress, a new myelin was created by denervated Schwann cells.^38^ TEM cross‐section imaging analyses of the reproduced myelin in the two groups showed that remyelination was robust in the experimental group, while there were still many incomplete myelinated axons in the control group after 8 weeks of recovery. To comprehend precise differences between them, four myelination‐related genes were selected for the qRT‐PCR assay: MPZ and MBP for the main structural protein of myelin, MAG for the early biomarker of myelination, and Krox20 for the master regulator of this process.^39^ Results demonstrated that a similar level of MAG at the 2nd week and upregulation of Krox20 was measured in both groups, while the MPZ and MBP were dramatically downregulated in the noncapped group. It could be inferred that myelination was normally activated and the partly myelination was attributed to the insufficient myelin related protein formation. Therefore, the application of PDLLA/PRGD conduit prevents the painful neuroma formation through coordinating the inflammation response, collagen deposition, and demyelination process.

## 4 Conclusion

We have evaluated the effects of PRGD/PDLLA capping conduits on inhibiting the formation of painful neuroma by the analysis of behavior, histological structure, and relative gene expression. The results indicated that inflammatory response, autotomy score, and scar deposition were reduced in the conduit group. Moreover, the myelin morphology of nerve stump capped with the PRGD/PDLLA capping conduits was thick and compact in TEM observation and the relative genes of myelin maturity were also upregulated. These factors contribute to the inhibition of painful neuroma. These changes may be due to the incorporation of RGD‐modified capping materials, which provided a favorable microenvironment for the nerve terminal recovery by inhibiting inflammatory amplification. Therefore, PRGD/PDLLA capping conduits could be a promising alternative used in capping nerve ends to inhibit neuroma formation.

## 5 Experimental Section

*Materials*: Poly(D,L‐lactic acid) (PDLLA) (*M*_w_: 250 000) was synthesized in house. Gly‐Arg‐Gly‐Asp‐Gly (RGD) and 1, l‐carbonyldiimidazole were purchased from GL Biochem (China). 1‐Ethyl‐3‐(3‐dimethylaminopropyl) carbodiimide, Masson's trichrome, and sirius red were purchased from Sigma‐Aldrich. Other chemicals were analytical grade. 3‐0 and 6‐0 monofilament nylon sutures were purchased from Ethicon, Inc. (Johnson & Johnson). SD rats were obtained from the Center for Disease Control and Prevention of Hubei Province (China). Epon 812 was purchased from Electron Microscopy Sciences (Hatfield, PA). Paraformaldehyde, paraffin, glutaraldehyde solution, osmium tetroxide, lead citrate, and uranyl acetate were purchased from Sinopharm Chemical Reagent Co., Ltd (China).

*Preparation of PDLLA/PRGD Capping Conduit*: The RGD‐modified poly{(lactic acid)‐*co*‐[(glycolic acid)‐alt‐(L‐lysine)]} (PRGD) was synthesized. First, (3S)‐3‐[4‐(benzyloxycarbonylamino) butyl] morpholine‐2, 5‐dione (BMD) was synthesized by bromoacetyl bromide and Ne‐(benzyloxycarbonyl)‐L‐lysine. Second, poly{(lactic acid)‐*co*‐[(glycolic acid)‐alt‐(Ne‐benzyloxycarbonyl‐L‐lysine)]} was obtained by copolymerization of D,L‐lactide and BMD. Then, poly{(lactic acid)‐*co*‐[(glycolic acid)‐alt‐(L‐lysine)]} (PLGL) was synthesized by the catalytic hydrogenation of PLGL. Finally, PLGL was modified with RGD peptide. PRGD (0.05 g) and PDLLA (0.95 g) were dissolved in ethyl acetate (5 wt%). The PRGD/PDLLA membrane was produced by a model mold and then rolled to fabricate PRGD/PDLLA capping conduit with one end closed. The final length of the capping conduit was 10 mm with an inner diameter of 2 mm and a tube wall thickness of 200 µm. The nerve capping conduits were sterilized with ultraviolet light for subsequent experiments and implantations.^40^

**Figure 7:**
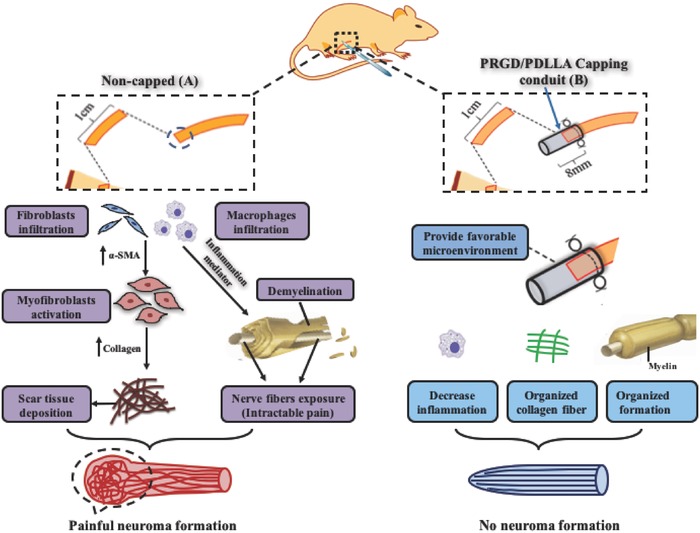
Schematic of PRGD/PDLLA conduit in preventing traumatic neuroma. A) As the consequence of peripheral nerve injury without capping, inflammatory cells (such as macrophages) accumulate at the injured site and secrete inflammatory mediators, which are able to develop hyperalgesia and induce adjacent fibroblasts to differentiate into myofibroblasts, which has the ability of spontaneous contraction. Then, prolonged inflammation response will lead to over expression of Type I collagen, which further produces mechanical stress to axons. This disturbs the recovery progress from Wallerian degeneration to remyelination; the exposed nerve fibers also attribute the intractable painfulness, leading to neuroma. B) With the covering of PRGD/PDLLA tube, a suitable microenvironment can be established for coordinating the apoptosis and tissue reconstruction, which directly isolates ectopic stimulation and prevents neuroma formation.

*Rat Model and Surgical Procedure*: Experiments undertaken in this study were approved by the Animal Care and Use Committees of Wuhan University of Technology and conformed to NIH guidelines. Adult Sprague‐Dawley rats (180–200 g) were randomly divided into two groups each with 12 rats: Group A: noncapped control group; Group B: PRGD/PDLLA capped group. Each rat was anesthetized with an intraperitoneal injection of 50 mg kg^−1^ body weight pentobarbital sodium. The right sciatic nerve was exposed and sharply transected into proximal and distal segments at the center of the right thigh. In the control group A, the proximal nerve stump was put aside without any treatment, but the distal stump was removed for 1 cm to avoid spontaneous nerve regeneration. In group B, the proximal stump was sutured with 6–0 monofilament nylon sutures to a depth of 2 mm into the PRGD/PDLLA capping conduit; the distal stump was also removed as in group A. In both groups, the muscle and skin layers were re‐approximated with 3–0 nylon sutures (**Figure** **7**).

*Behavioral Observation*: Autotomy scores were recorded once a week after surgery. The extent of autotomy was assessed using the modified point scale.^41^ One point was assigned to an injury of one or more nails, and one point was added if each distal‐half toe was injured; then, one extra point was added for each proximal‐half of an injured toe. Finally, one point was assigned for autotomy of metatarsus and tarsal area, respectively. The maximum score for this scale was 13 points.

*Histological Evaluation*: The proximal nerve segments were obtained from each group for the histological analysis on 2 and 8 weeks' postsurgery. To evaluate the collagen deposition of the proximal stumps, the specimens were fixed with paraformaldehyde (4 wt%), and then were dehydrated with ethanol, embedded in paraffin, cut into 5 µm thickness, and stained with Masson's trichrome and sirius red for imaging by inverted microscopy (Olympus, IX71, Japan). To analyze myelination morphology, the nerve specimens were fixed in glutaraldehyde solution (2.5% v/v). The samples were postfixed with osmium tetroxide (1% w/w) for another 2–3 h, dehydrated by graded ethanol, embedded in Epon 812, and sliced to 60 nm. The sections were stained with lead citrate and uranyl acetate, and observed by a TEM (TecnaiG220 TWIN, FEI, USA). Photographs of each nerve sample were quantitatively analyzed by ImageJ software (v1.51n, NIH).

*Immunohistochemical Analyses*: The nerve paraffin sections of proximal stumps were used to detect T lymphocyte and macrophage via CD3 and CD68 immunohistochemical staining. The nerve paraffin sections were cut into 5 mm thickness, dewaxed in xylol, and dehydrated in ethanol, followed by blocking with goat serum at 24 °C for 30 min. The sections were, respectively, incubated in a solution containing anti‐CD3 primary antibody (1:1000 dilution; Abcam) and anti‐CD68 (1:1000 dilution; Abcam) at 4 °C overnight, and subsequently incubated with biotinylated secondary antibodies, respectively, for 20 min at 37 °C and visualized by diaminobenzidine. Nuclei were counterstained with hematoxylin, and the nerve sections were imaged under an inverted microscope. The positive number of CD3 and CD68 was analyzed by an image analysis system.

*Quantitative Real‐Time PCR*: At the 2nd and 8th weeks after implantation, proximal nerve segments were collected, and changes in the transcription of genes α‐SMA, Krox20, Mag, Mbp, Mpz, TNF‐α, and IL‐1β were studied via qRT‐PCR.^42^ The total RNA of each sample was extracted using Trizol reagent (Invitrogen) and reverse transcribed into cDNA. All the primers used in the qRT‐PCR analysis are listed in **Table** **1**. The thermal cycling program was: 10 min denaturation at 95 °C; for amplification and quantification 40 cycles of 15 s at 95 °C, 60 s at 60 °C, and 20 s at 75–95 °C. The mix without template was treated as blank control and each reaction was run in triplicates. The qRT‐PCR was carried out and conducted with a real‐time PCR system (Applied Biosystems 7300 Fast, Thermo Fisher Scientific). The relative quantification of gene expression was determined by 2‐ΔΔ*C*_t_ method.^43^

**Table 1:** Primer sequences of the genes for qRT‐PCR analysis

Gene name	Forward sequence (5ʹ‐3ʹ)	Reverse sequence (5ʹ‐3ʹ)	Product size [bp]
α‐SMA	GCTCCTCCAGAACGCAAATAT	GGGCCAGCTTCGTCATACTC	110
Krox20	GCTACCCAGAAGGCATCATCA	GAGTAGAGGTGGTCCAGTTCAGG	178
MAG	CCCTGCCTCTGTTTTGGATA	CGGGTAGTTCTTGGGGTAGG	194
MBP	ATGGGAAACCACTCTGGAAAG	AGTTATTCTTTGGGTCTGCTGTG	229
MPZ	GTGGTTTACACGGACAGGGAA	CCTTGGCATAGTGGAAGATTGA	163
TNF‐α	CTTCTGTCTACTGAACTTCGGGGT	ATCTGAGTGTGAGGGTCTGGGC	104
IL‐1β	TGACCTGTTCTTTGAGGCTGAC	CATCATCCCACGAGTCACAGAG	272
GAPDH	TTCCTACCCCCAATGTATCCG	CATGAGGTCCACCACCCTGTT	281
Actin	TGCTATGTTGCCCTAGACTTCG	GTTGGCATAGAGGTCTTTACGG	240

*Statistical Analysis*: Experimental data were processed using PASW Statistics 18 software (SPSS Inc., Chicago, IL, USA). All numerical data were expressed as mean ± standard deviation. T‐test was used to determine the significance of the differences between the groups. *P* < 0.05 was considered as statistically significant.

## Conflict of Interest

The authors declare no conflict of interest.
